# Subicular neurons represent multiple variables of a hippocampal-dependent task by using theta rhythm

**DOI:** 10.1371/journal.pbio.3001546

**Published:** 2022-01-31

**Authors:** Su-Min Lee, Jae-Min Seol, Inah Lee

**Affiliations:** Department of Brain and Cognitive Sciences, Seoul National University, Seoul, Korea; Center for Brain Research, Medical University of Vienna, AUSTRIA

## Abstract

The subiculum is positioned at a critical juncture at the interface of the hippocampus with the rest of the brain. However, the exact roles of the subiculum in most hippocampal-dependent memory tasks remain largely unknown. One obstacle to make comparisons of neural firing patterns between the subiculum and hippocampus is the broad firing fields of the subicular cells. Here, we used spiking phases in relation to theta rhythm to parse the broad firing field of a subicular neuron into multiple subfields to find the unique functional contribution of the subiculum while male rats performed a hippocampal-dependent visual scene memory task. Some of the broad firing fields of the subicular neurons were successfully divided into multiple subfields similar to those in the CA1 by using the theta phase precession cycle. The new paradigm significantly improved the detection of task-relevant information in subicular cells without affecting the information content represented by CA1 cells. Notably, we found that multiple fields of a single subicular neuron, unlike those in the CA1, carried heterogeneous task-related information such as visual context and choice response. Our findings suggest that the subicular cells integrate multiple task-related factors by using theta rhythm to associate environmental context with action.

## Abstract 2

The subiculum is positioned at the interface between the hippocampus and the rest of the brain, but what is its role in hippocampal-dependent memory tasks? This neurophysiological study provides novel insights into how the subiculum represents multiple cognitive variables by using both rate and temporal codes.

## Introduction

The hippocampal formation plays key roles in fundamental cognitive functions, including spatial navigation and episodic memory [1–3]. The subiculum, a region within the hippocampal formation, has long been considered the area from which cortical outputs of the hippocampus emanate [4,5]. However, viewing the subiculum as an area that passively transmits hippocampal information to cortical regions might be inappropriate, because the subiculum is connected not only with the CA1 of the hippocampus but also with other areas, including the medial prefrontal cortex, entorhinal cortex, retrosplenial cortex, perirhinal cortex, postrhinal cortex, nucleus accumbens, basal amygdala, and various subcortical regions [6–8].

Physiologically, it has been reported that the neural correlates of the subiculum are significantly different from those of the CA1 during spatial navigation. Specifically, neurons in the subiculum tend to exhibit broader place fields than those in the CA1 [9–12]. Also, place cells in the subiculum are more attuned to movement-related factors, such as direction and motion, during navigation compared with CA1 place cells [13–16]. A few studies have also suggested that the subiculum is essential in remembering places and environmental contexts [17–21]. However, the exact roles of subicular neurons, especially in a goal-directed memory task, still remain largely unknown.

In our previous study, we reported that neurons in both the subiculum and CA1 showed rate remapping according to task-related factors, specifically visual scene and choice response in a visual scene memory (VSM) task. In the VSM task, rats were required to make choices in a T-maze using the visual scene stimulus presented around the maze [11]. Interestingly, place cells in the CA1 showed such firing properties while coding very specific locations in space, whereas cells in the subiculum fired similarly while mapping broader areas (e.g., entire stem or choice arm region), as if they represented the cognitive structure of the task by schematically parsing the environment. On the basis of these results, we speculated that position-linked environmental information in the hippocampus in the VSM task [22,23] might be translated into contextual action–related information that can be communicated with other brain regions.

One major obstacle that poses great difficulties for investigations of the neural correlates of subicular neurons is their higher spontaneous firing rates and broader firing fields in space compared with those of place cells in the hippocampus [10–12]. These firing characteristics of subicular neurons make it difficult to apply the conventional analytical techniques optimized for place cells recorded from hippocampus. In the hippocampus, such techniques work well because place fields are more restricted to specific locations of the environment with a higher signal-to-noise ratio compared with the subiculum. For example, in our previous study [11], we sought to identify field boundaries of subicular cells by finding local minima through statistical comparisons of trial-by-trial firing rates between neighboring bins. However, such methods had shortcomings, such as defining some subicular cells as having no fields and ignoring small subfields in the presence of a more dominant field with a higher firing peak.

Notably, some previous studies attempted to parse the broad spatial firing field of a subicular neuron into smaller fields using the phases of spikes in relation to theta rhythm [10,24]. Here, inspired by these studies, we compared the traditional rate–based field detection method with the theta phase–based field detection method using the same physiological data recorded from the CA1 and subiculum in our previous study [11]. The current study showed that the phase-based analysis could successfully parse subicular firing fields into multiple subfields and that these newly parsed place fields in the subiculum represented task-related information better. Importantly, some subicular cells represented multiplex information associated with the VSM task through their phase-based subfields, possibly suggesting a unique role of the subiculum in integrating environmental information with action.

## Results

### Electrophysiological recording in the subiculum and CA1 in the VSM task

**Fig 1:**
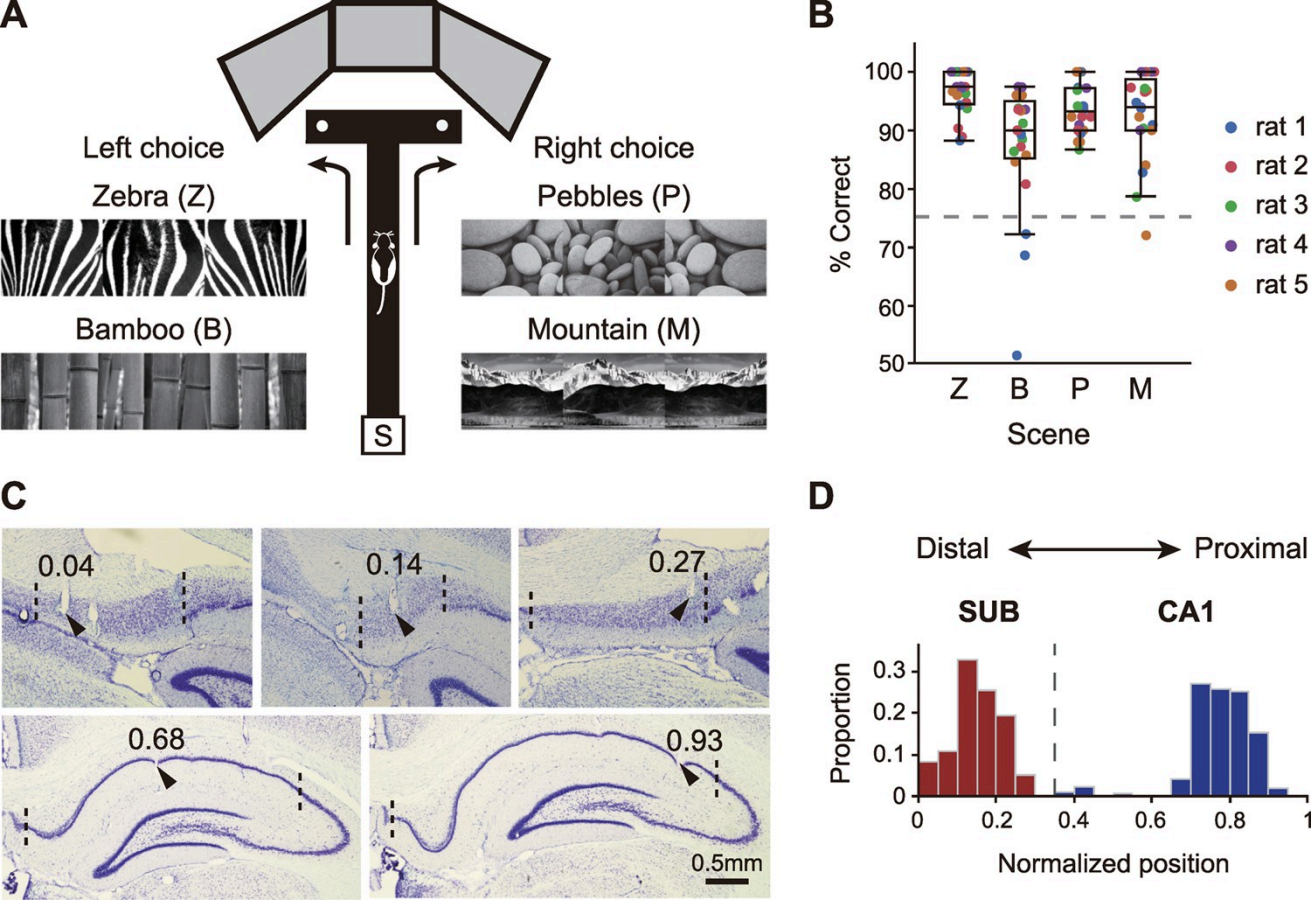
Behavioral task and histological verification of electrophysiological recordings. (**A**) VSM task. As a trial begins, the rat runs out onto the track of a T-maze from the start box (S), and one of 4 visual scene stimuli (Zebra, Z; Bamboo, B; Pebbles, P; Mountain, M) is presented on LCD monitors. Each scene stimulus is associated with either the left or right arm of the T-maze. (**B**) Behavioral performance during recording sessions (21 sessions from 5 rats). Each dot corresponds to the percent correct for each scene stimulus of a session and is color-coded for individual rats. Box plot indicates interquartile range and median value. The median values exceeded the performance criterion (dashed line, 75%) for all scenes. (**C**) Photomicrographs of Nissl-stained coronal brain sections with verified electrode tips (black arrows). Numbers above the arrows indicate normalized positions of marked recording sites along the proximodistal axis. Dashed lines represent the anatomical boundaries of the CA1 or subiculum. Upper and lower rows show recording sites from the subiculum and CA1, respectively. (**D**) Proportional distribution of cells recorded in the CA1 (blue) and SUB (red) along the proximodistal axis (CA1, *n =* 270; SUB, *n* = 151). The positions are normalized to account for differences in relative length between 2 regions. The dashed line at 0.36 indicates the boundary between 2 regions. Data associated with this figure can be found in S1 Data file. SUB, subiculum; VSM, visual scene memory.

In the VSM task, rats (*n =* 5) learned to associate each scene stimulus with either a left or right turn response on the T-maze (**Fig 1A**). During recording sessions, rats performed the VSM task well above performance criterion (75%) for all stimuli (*p*-values < 0.0004 for all scenes, one-sample Wilcoxon signed-rank test; **Fig 1B**). Tetrodes located at the boundaries of either the CA1 or subiculum (including the border between them) were excluded from the analysis (**Fig 1C**). To quantify the anatomical distributions of recording locations for the CA1 and subiculum along the proximodistal axis, we measured the relative positions from which individual cells were recorded and normalized them across rats (**Fig 1D**). Only complex-spiking cells satisfying our unit-filtering criteria (CA1, *n =* 270; subiculum, *n* = 151; see **Methods**) were used for analysis. Subicular cells were found along the entire proximodistal axis, whereas CA1 cells were mainly recorded from the intermediate to proximal portions of the CA1. More details can be found in our previous study [11].

### Limitations of the firing rate–based method in detecting place fields in the subiculum

**Fig 2:**
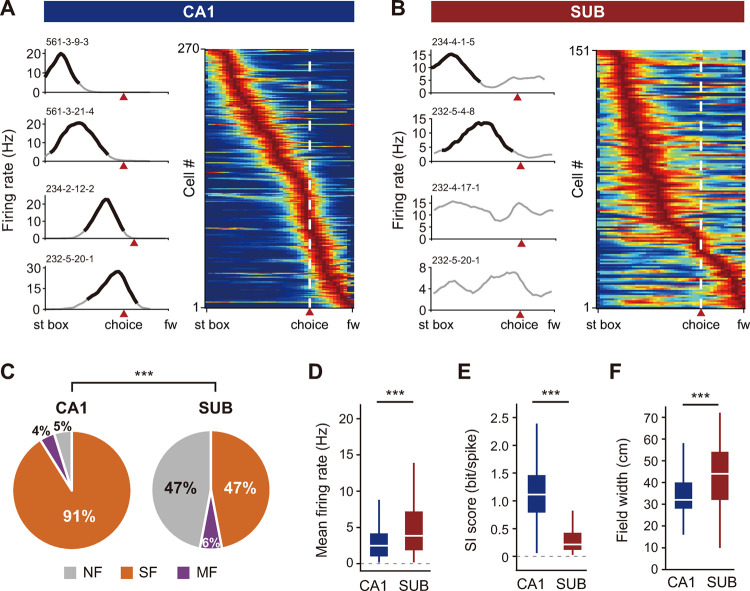
Poorer spatial firing patterns in the subiculum than the CA1. (**A**, **B**) Firing rate maps of single cells (left) and cell populations (right) in the CA1 (A) and subiculum (B), plotted as a function of the linearized position on the T-maze from the start box to the food well in both arms. Red arrowheads indicate the choice point after which rats’ positions diverged between the left and right choice trials. On the firing rate maps of single cells, legitimate place fields are overlaid with thick black lines, and non-place fields that did not pass the place field criteria are marked by gray lines. Serial numbers on the upper left corner are cell IDs. Population firing rate maps are sorted according to peak firing rate of each cell on the T-maze. White dashed lines and red arrowheads indicate the choice points. (**C**) Proportional differences of place cells between the subiculum and CA1, defined by the firing rate–based method. Cells are classified into 3 groups according to the number of place fields per cell: “SF” for one field, “MF” for more than one field, and “NF” for no field. ***p < 0.0001. (**D–F**) Differences in mean firing rate (D), SI score (E), and place field width (F) of recorded cells between the CA1 and subiculum. ***p < 0.0001. Data associated with this figure can be found in S1 Data file. fw, food well; MF, multi-field; NF, non-place field; SF, single-field; SI, spatial information; st box, start box; SUB, subiculum.

Prior studies [9–12] reported that cells in the subiculum fire at higher rates with lower spatial selectivity than those in the CA1, a finding also confirmed in our study. That is, cells recorded from the CA1 fired at focal and restricted locations along the T-maze (**Fig 2A**), whereas cells recorded from the subiculum tended to show broad and continuous firing fields (**Fig 2B**), making it challenging to identify a place field using the conventional field detection method based on spatial firing rates. Specifically, although some subicular cells exhibited spatially tuned place fields similar to CA1 place fields (cells 234–4–1–5 and 232–5–4–8 in **Fig 2B**), some background spiking activity continued to occur outside their place fields. Furthermore, other subicular cells fired continuously across the entire track (cells 232–4–17–1 and 232–5–20–1 in **Fig 2B**), complicating efforts to define the field boundaries for these cells. These differences in field characteristics between the CA1 and subiculum can be more clearly observed in population rate maps constructed by stacking the rate maps of individual cells (**Fig 2A** and **2B**).

To quantitatively compare differential firing patterns between the 2 regions, we first classified cells according to the number of place fields: no place field, a single field, or multiple fields. A spatial firing distribution was considered a place field if its peak firing rate exceeded 1 Hz and its spatial information content (bits/spike) exceeded 0.5. Field boundaries were set at the spatial bins in which the associated firing rates dropped below 33% of the peak firing rate (see **Methods**). Of cells that were active during the rat’s outbound journey on the T-maze, approximately 90% were single-field cells in the CA1, while only about half of cells exhibited either single- or multi-fields in the subiculum ($\chi_{\left(1\right)}^{2}$ = 122.96, *p* < 0.0001; chi-squared test; **Fig 2C**). With respect to basic firing properties, cells in the CA1 showed lower firing rates (Z = 5.14, *p* < 0.0001; **Fig 2D**) with higher spatial information (Z = 14.2317, *p* < 0.0001; Wilcoxon rank-sum test; **Fig 2E**), compared with those in the subiculum. Overall, as we reported previously (Lee and colleagues, 2018), subicular cells exhibited less spatial tuning than CA1 cells (spatial selectivity: 4.04 ± 0.08 in CA1, 2.24 ± 0.07 in subiculum; sparsity: 0.41 ± 0.01 in CA1, 0.74 ± 0.17 in subiculum; mean ± SEM; *p*-values < 0.0001; Wilcoxon rank-sum test). In addition, field width was larger in subicular place cells than those in CA1 (Z = 5.96, *p* < 0.0001; Wilcoxon rank-sum test) for both single- and multi-field cells (**Fig 2F**). Taken together with the comparisons made between the 2 regions based on other traditional measures in our previous study [11], these spatial firing patterns made it difficult to define place fields for individual neurons in the subiculum compared with those in the CA1.

### Identification of latent place fields based on theta phase precession of spiking

Our findings show that the fundamental differences in spatial firing characteristics between the CA1 and subiculum make it difficult to use conventional approaches commonly employed for analyzing place fields in both regions because these approaches have mostly been developed for place fields of cells in the hippocampus and not for those in the subiculum. In fact, a large number of subicular cells that would have been defined as no-field cells by conventional field detection methods did fire more vigorously at particular locations of the track (**Fig 2B**, cells 232–4–17–1 and 232–5–20–1). However, the conventional field detection algorithm was unable to detect such spatial firing patterns because of the higher spontaneous firing activities throughout the track in subicular cells compared with CA1 neurons. Our previous study tried to locate field boundaries in these cells by adjusting the threshold for detecting field boundaries or by finding local minima through statistical comparisons of trial-by-trial firing rates between neighboring bins. However, such methods still defined some subicular cells as having no field. Furthermore, the conventional field detection algorithm tended to ignore a small subfield if there was one dominant field with a very high firing peak.

**Fig 3:**
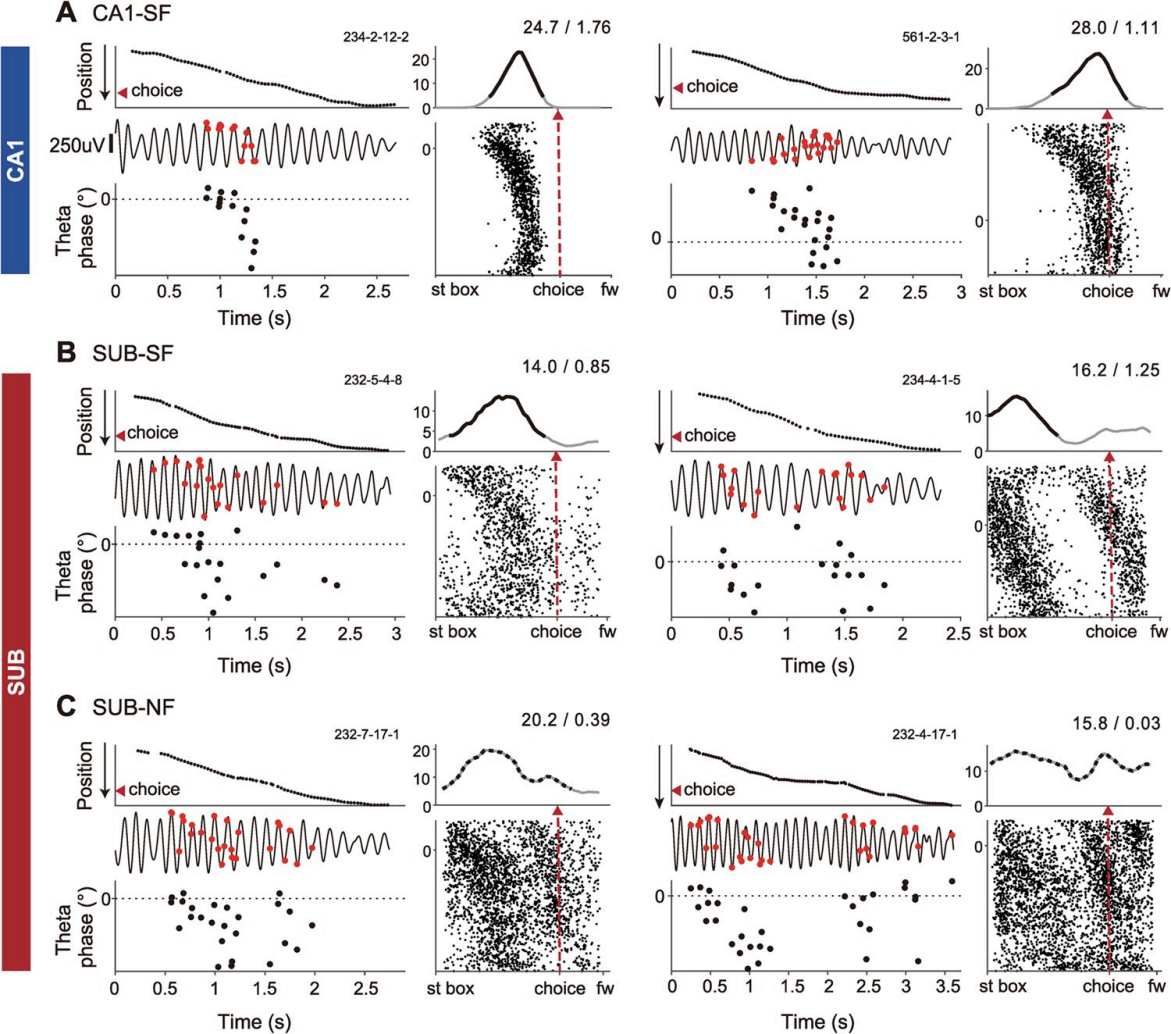
Robust multiple theta phase precessions in the subiculum. (**A–C**) Representative examples of theta phase precession in the single-field cells in CA1 (A), subiculum (B), and non-place field cells in the subiculum (C). The left column for each cell consists of linearized position (top), a raw trace of theta oscillation (middle), and spiking theta phases (bottom) in the temporal axis in a single trial. Spikes in the raw theta traces are marked by red circular dots. Scale bar, 250 *μ*V. Spiking theta phases are plotted within a range of 360°, and the initial phase is adjusted for clear observation of theta phase precession. Serial numbers in the upper right corner are cell IDs. The right column displays a linearized firing rate map (top) and a position phase plot (bottom) on the spatial axis across a session. Black solid lines overlaid on the firing rate maps indicate verified place fields, whereas black dotted lines are non-place fields. Numbers above firing rate maps denote peak firing rates (Hz) and spatial information scores (bit/spike) of place or non-place fields. Red arrowheads and red dashed lines mark choice points. Note that subicular cells showed multiple cycles of theta phase precession, some of which were as robust as those of CA1 cells. fw, food well; NF, non-place field; SF, single-field; st box, start box; SUB, subiculum.

To overcome such limitations, we explored the possibility of defining place fields using theta phase precession, a well-known phenomenon in which theta-related phases of spikes of a neuron gradually shift to earlier phases as the rat repeatedly passes through the cell’s place field [25,26]. In particular, we examined whether the broad firing field of a subicular neuron could be divided into multiple subfields if it were defined by theta phases of spikes. As shown in **Fig 3**, theta phase precession occurred robustly within the identified unitary place field in both the CA1 and subiculum as the rat ran along the track (CA1 single-field cells 234–2–12–2 and 561–2–3–1 in **Fig 3A**; subicular single-field cells 232–5–4–8 and 234–4–1–5 in **Fig 3B**). Importantly, those cells previously classified as having no place field exhibited multiple cycles of robust theta phase precessions in the subiculum (subicular non-place field cells 232–7–17–1 and 232–4–17–1 in **Fig 3C**).

**Fig 4:**
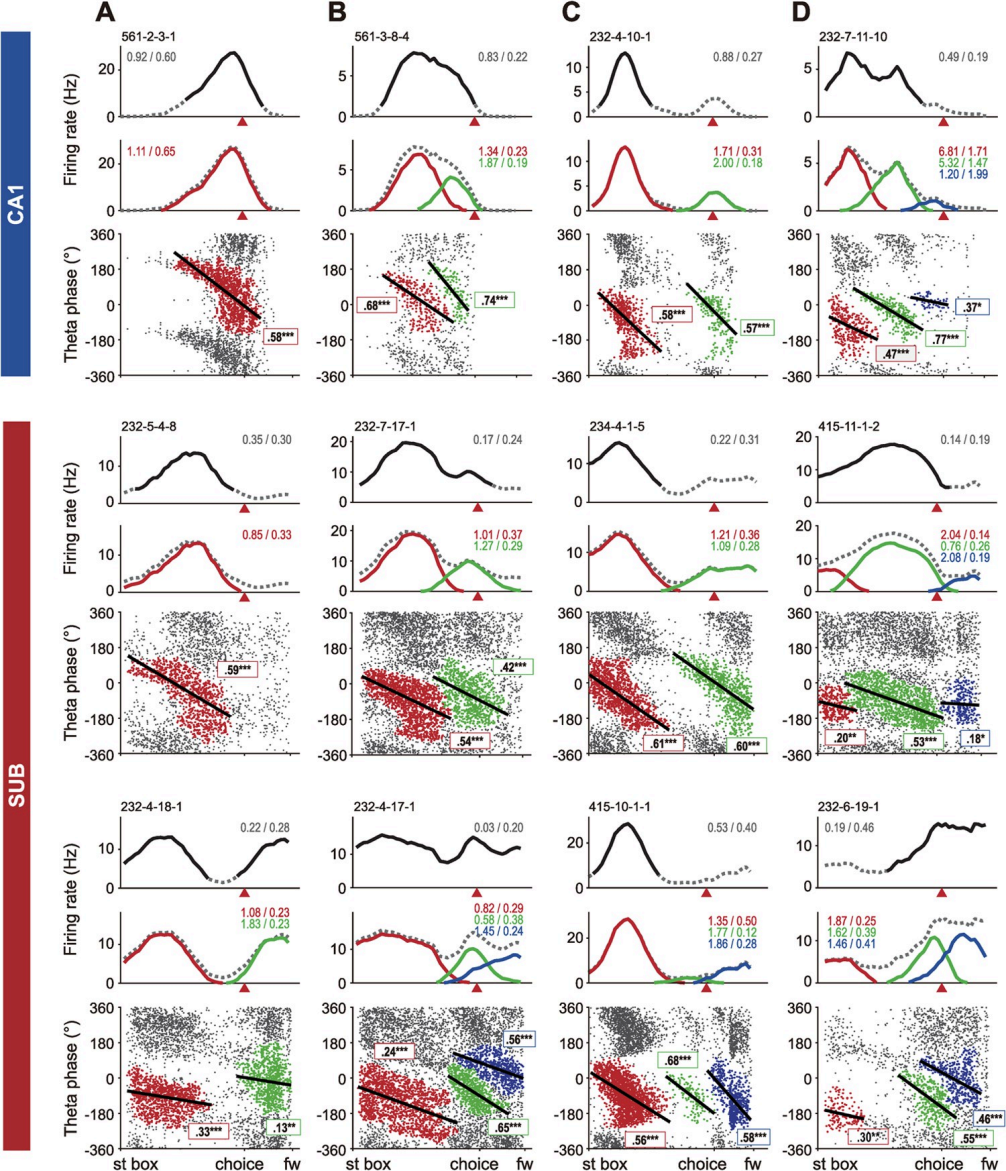
Identification of place fields based on spiking theta phases in the CA1 and subiculum. (**A–D**) For each cell example, a linearized firing field based on firing rates (rate-based field indicated by solid black line; top), linearized firing fields based on theta phases (phase-based fields denoted by different colors; middle), and a position phase plot on the spatial axis across a session (bottom) are shown. Gray dotted line indicates the mean firing field. The numbers on the right corner indicate spatial information scores using firing rates (bit/spike; front) and spiking theta phases (bit/cm; back) obtained from the entire firing activities of a cell (gray) or individual phase-based fields (color coded). Red arrowheads denote choice points. Serial numbers above the firing rate maps are cell IDs. Spike clusters in position phase plots are color-coded with the same colors used for the firing rate maps. Black straight lines on spike clusters indicate the circular–linear regression lines. The numbers and asterisks in the box with colored borders are circular–linear correlation coefficients and their significance for phase-based fields in the same color. *p < 0.05, **p < 0.01, ***p < 0.0001. fw, food well; st box, start box; SUB, subiculum.

To identify a spike cluster that belonged to a single theta precession cycle in the phase position plot, we used the DBSCAN (Density-Based Spatial Clustering with Applications of Noise) algorithm (see **Methods** for details). We compared the results of two different methods for detecting place fields: a firing rate–based method that finds a “rate-based field”, and the theta phase precession–based DBSCAN method, which finds a “phase-based field”. Both algorithms produced the same results in some cells in both the CA1 and subiculum (**Fig 4A**). However, we were also able to find new place fields for other cells based on the phase-based method. Specifically, some cells that were originally classified as single-field cells were converted into multi-field cells by application of the theta phase–based clustering algorithm (**Fig 4B**–**4D**). That is, in some cells, existing rate-based fields were subdivided into more than 2 phase-based fields (**Fig 4B**). In other cells, additional place fields that might not have been detectable by the rate-based method (mostly owing to low firing peaks) were revealed by the phase-based clustering (**Fig 4C**). In a final group of cells, the phase-based method separated an existing field and added a new field at the same time (**Fig 4D**).

**Fig 5:**
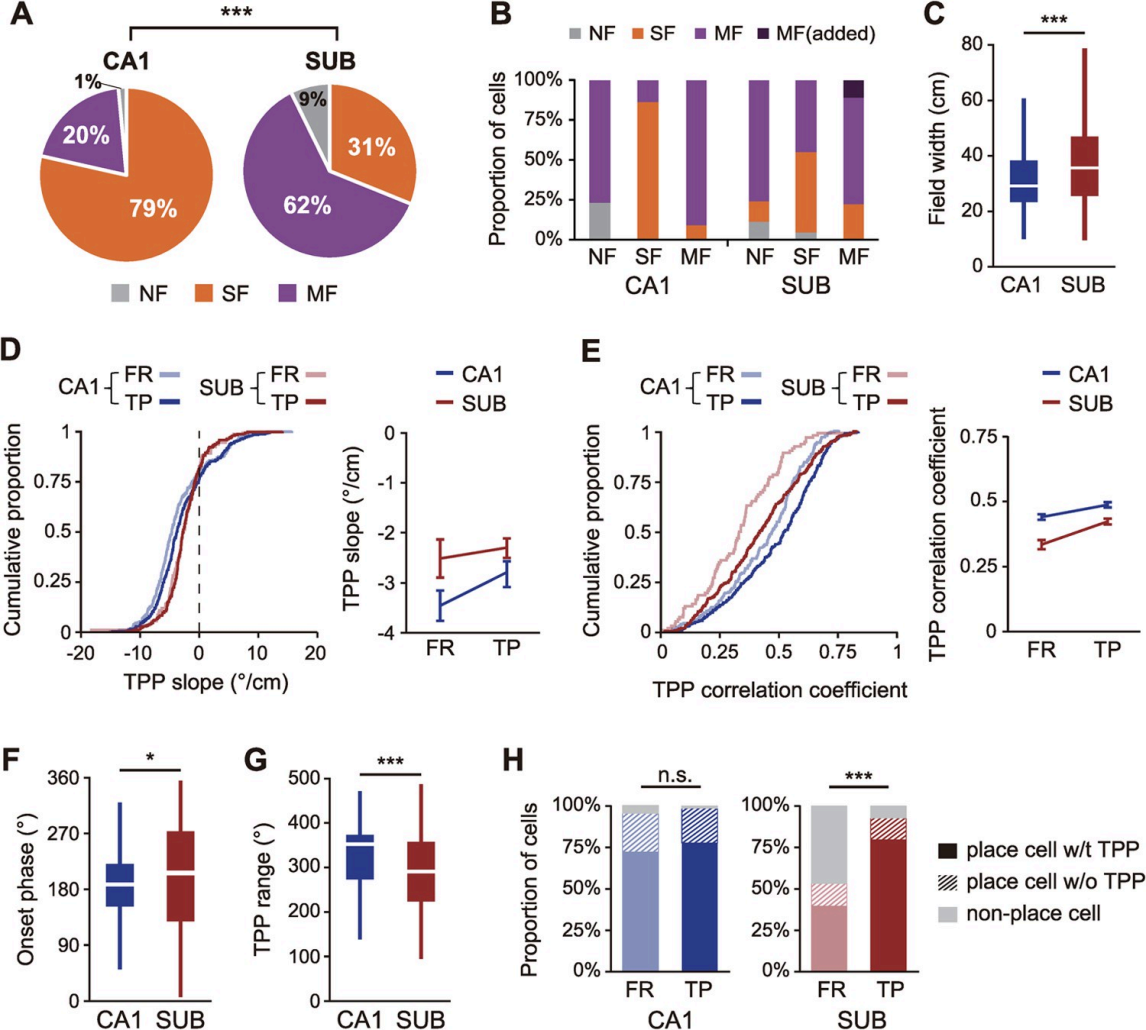
Advantages of theta phase–based field detection. (**A**) Difference between the CA1 and subiculum in the proportion of place cells associated with different numbers of place fields, when defined using the theta phase–based method. Cells are classified into 3 groups: “SF” for one field, “MF” for more than one field, and “NF” for no field. ***p < 0.0001. (**B**) Categorical changes of cells within each rate-based cell group (NF, SF, and MF on the *x* axis) as the field identification method was shifted to the one using theta phase. The bar graph shows the proportion of cells in each cell group (classified by the rate-based method) was recategorized after the phase-based method. (**C**) Regional differences in place field width after phase-based field detection. ***p < 0.0001. (**D–E**) Cumulative distributions of TPP slope (D) and correlation coefficient (E) of place cells for each method (rate-based, FR, and theta phase-based, TP) and each region. Line graphs on the right side of each panel display mean values and standard errors for the same data. (**F–G**) Regional differences in the onset phase (H) and the phase shift range (G) of the TPP. These measurements were obtained only from the phase-based fields that showed significant TPP. *p < 0.05, ***p < 0.0001. (**H**) Changes in the proportion of place cells that exhibited significant TPPs (w/t TPP) when the fields were identified by the conventional rate–based method (FR) versus the theta phase–based method (TP) in the CA1 and subiculum. ***p < 0.0001. Data associated with this figure can be found in S1 Data file. FR, firing rate; MF, multi-field; NF, non-place field; SF, single-field; TP, theta phase; TPP, theta phase precession.

The proportions of cells showing different numbers of place fields changed when using the phase-based clustering method compared with the rate-based method. Specifically, phase-based clustering classified 20% of CA1 cells and 62% of subicular cells as multi-field cells and only 9% of subicular cells as having no field ($\chi_{\left(1\right)}^{2}$ = 106.70, *p* < 0.0001; chi-squared test; **Figs 5A** and **S1**). When the categorical changes were examined for each cell group, it turned out that three-quarters of the rate-based non-place cells in the CA1 and subiculum exhibited multiple phase-based place fields (**Fig 5B**). In addition, some rate-based single-field cells in the CA1 (14%) and subiculum (45%) were converted to multi-field cells by the phase-based clustering. We also found that some rate-based multi-field cells in the subiculum exhibited additional phase-based fields after applying the phase-based protocol (“MF-added” in **Fig 5B**). Although the widths of phase-based place fields remained still significantly larger in the subiculum than in the CA1 (Z = 4.08, *p* < 0.0001; Wilcoxon rank-sum test; **Fig 5C**), other firing properties of individual fields defined by theta phase became comparable between the 2 regions. In particular, when we compared the 2 regions by both rate-based spatial information (bit/spike) and phase-based spatial information (bit/cm) measured for all spiking activities associated with outbound journeys in individual neurons (see **Methods** for details), the distributions were well separated between the CA1 and subiculum (**S2A Fig**). However, when the same distributions were obtained by using only the spiking activities within the boundaries of the phase-based fields, the 2 distributions of CA1 and subiculum largely overlapped (**S2B Fig**). These findings suggest that the place cells in both regions may share more common firing properties than previously thought when the place fields are defined by theta phases of spikes instead of the conventional rate–based method.

We next examined the robustness of theta phase precession of place cells in the subiculum compared with that in the CA1 using circular statistics (linear regression and linear correlation) for each spike cluster [27]. We found that the slope of theta phase precession was significantly different between the 2 regions (F_(1,435)_ = 4.43, *p* = 0.036), but it was not affected by the field–identification method (F_(1,613)_ = 0.59, *p* = 0.44, two-way mixed ANOVA with region as the between-subject factor and the field identification method as the within-subject factor; **Fig 5D**). There was no interaction between the region and field-detection method (F_(1,613)_ = 0.52, *p* = 0.47), mostly attributable to the reduced regional difference when the phase precession slope was calculated based on the phase-based fields compared to the rate-based fields. The precession slope of rate-based fields tended to be steeper in the CA1 than in the subiculum (t_(805)_ = 2.01, *p* = 0.045 for Bonferroni-corrected unpaired two-sample *t* test; corrected α = 0.0125), an outcome that could be expected based on the larger field width of subicular place cells. However, the regional difference in slope diminished when using the phase-based method (t_(496)_ = 1.64, *p* = 0.102). The slope of theta phase precession was not affected by the field detection method within each region (CA1, t_(555)_ = 1.39, *p* = 0.16; SUB, t_(637)_ = 0.03, *p* = 0.98). On the other hand, the strength of theta phase precession of place cells evaluated by circular–linear correlation coefficient was significantly different between the CA1 and subiculum (F_(1,445)_ = 31.57, *p* < 0.0001) and between the 2 field detection methods (F_(1,655)_ = 50.25, *p* < 0.0001; two-way mixed ANOVA; **Fig 5E)**. The interaction between the region and method was not significant (F_(1,655)_ = 3.68, *p* = 0.055). Post hoc tests revealed that the phase precession strength increased in phase-based fields compared with rate-based fields in both regions (CA1, t_(565)_ = 4.77, *p* < 0.0001; subiculum, t_(695)_ = 5.36, *p* < 0.0001; unpaired two-sample *t* test with Bonferroni correction; corrected α = 0.0125). Although precession strength was significantly lower in the subiculum than in the CA1 even based on the phase-based field detection (rate-based, t_(896)_ = 5.08, *p* < 0.0001; phase-based, t_(541)_ = 4.03, *p* < 0.0001), the precession strength in the subiculum increased closer to that of the CA1.

To further compare the basic properties of theta phase precession between CA1 and subiculum, we screened for the phase-based fields that showed significant theta phase precession based on the following criteria: (i) the range of phase shift ≥90°; (ii) the slope of circular–linear regression line <0; and (iii) *p*-value of circular–linear correlation ≤0.05. In both CA1 and subiculum, 70% of phase-based fields exhibited significant theta phase precession by meeting all 3 criteria (CA1, *n =* 235/337; subiculum, *n* = 212/300). In the subiculum, the onset phase was slightly, yet significantly, earlier (Z = 2.81, *p* = 0.005; **Fig 5F**) and the range of phase shift was significantly smaller (Z = 4.48, *p* < 0.0001, Wilcoxon rank-sum test; **Fig 5G**) than in CA1. The proportion of place cells showing significant theta phase precession increased in the subiculum as the phase-based method was applied compared to using the conventional rate–based method ($\chi_{\left(1\right)}^{2}$ = 61.33, *p* < 0.0001; chi-squared test; **Fig 5H**), but this was not the case in the CA1 ($\chi_{\left(1\right)}^{2}$ = 4.95, *p* = 0.084). These findings indicate that the field detection method based on theta phase precession of spikes effectively identified multiple subfields enveloped in the broad firing activities of the subicular cells.

### Increase in task-relevant information in phase-based fields of subicular neurons

**Fig 6:**
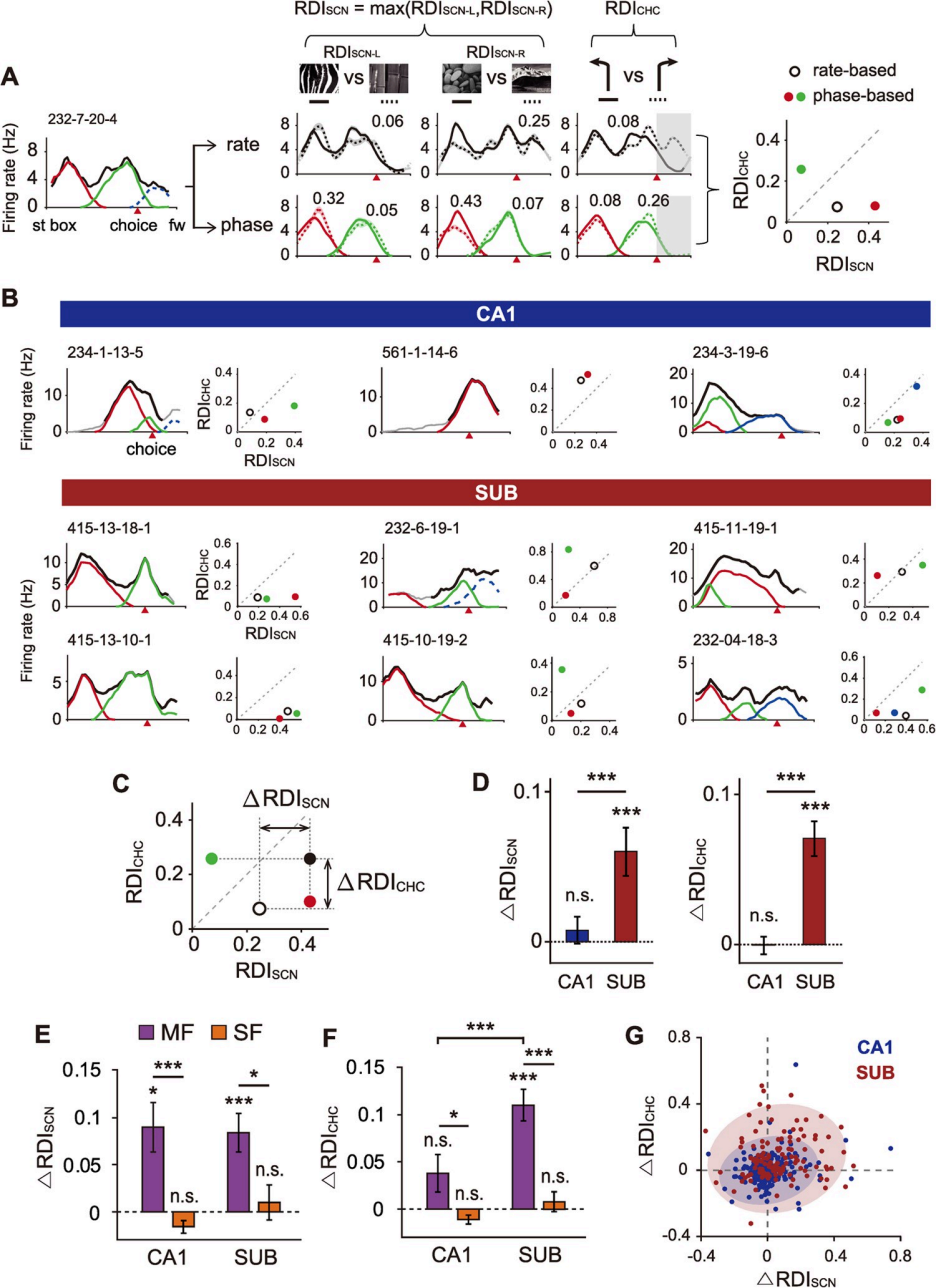
Scene- and choice-dependent rate remapping is enhanced in the subiculum but not in the CA1 based on the phase-based field detection. (**A**) A representative subicular cell illustrating how different indices for scene (RDI_SCN_) and choice (RDI_CHC_) are obtained. The linearized firing rate map in the left panel shows rate-based fields and phase-based fields averaged across all trials. Middle panel shows the firing rate maps associated with different task-relevant information. Rate-based fields are marked as black lines (upper row), and phase-based fields are color-coded (bottom row). Shaded areas overlaid on the fields are standard errors. Numbers above the fields indicate RDI values. The rightmost panel shows RDI_SCN_ and RDI_CHC_ values for individual fields marked as dots on the scatter plot; open black dots correspond to rate-based fields and color-coded dots denote phase-based fields. (**B**) Example neurons in the CA1 and subiculum with their RDI values associated with scene and choice information. Within each neuron, its linearized firing rate map (left) and RDI scatter plot (right) are shown as in (A). Solid black lines on the firing rate maps are FR-based firing fields, and color-coded lines are TP-based place fields, with arm fields depicted in dotted lines. Serial numbers above the rate maps are cell IDs. Red arrowheads indicate choice points. (**C**) Illustration showing how RDI differences (i.e., ΔRDI_SCN_ and ΔRDI_CHC_) are measured using the rate-based method (open black circle) and phase-based method (closed black circle). The filled black circle is a representative point for the phase-based method, marked by selecting maximum values among RDIs obtained from all phase-based fields. (**D**) Bar graphs comparing the magnitude of changes in RDI_SCN_ and RDI_CHC_ between regions. Data are presented as means ± standard error of the mean. ***p < 0.0001. (**E–F**) Comparison of the magnitudes of RDI changes for scene (E) and choice (F) information between the cell groups with MFs and SFs within each region (CA1, *n =* 46/166; subiculum, *n* = 81/51; MF/SF). *p < 0.0125, ***p < 0.0001. (**G**) Scatter plot jointly displaying ΔRDI_SCN_ and ΔRDI_CHC_ for all neurons in the CA1 and subiculum. Colored ellipses indicate bivariate normal distributed regions (coverage 95%). Note that subicular neurons are more dispersed in the first and second quadrant than CA1 neurons. Data associated with this figure can be found in S1 Data file. FR, firing rate; MF, multi-field; RDI, rate difference index; SF, single-field; SUB, subiculum; TP, theta phase.

We previously reported that firing of neurons in the CA1 and subiculum was correlated with the visual scene stimulus and choice response in the VSM task in the form of rate remapping [11,22]. Here, we examined whether scene- or choice-dependent rate remapping also appeared in phase-based fields of neurons in the CA1 and subiculum. To quantify rate remapping, we obtained a rate difference index (RDI) for individual rate-based and phase-based fields using the firing rate maps associated with different task conditions (see **Methods**; **Figs 6A** and **S3**). The RDI for choice response (RDI_CHC_) was calculated using only the spiking activity recorded up to the choice point. In contrast, for the scene-based RDI (RDI_SCN_), spiking activity associated with places beyond the choice point were also included because only the scenes associated with the same choice arm were compared. Place fields representing only arm areas were excluded from the RDI analysis, and the cells having such arm fields only were excluded as well (CA1, *n =* 212; subiculum, *n* = 132).

We found that the phase-based field detection method extracted task-relevant information more clearly than the rate-based method, especially in the subiculum. It also revealed new information that went undetected by the conventional rate–based method. For example, the phase-based method identified 2 fields for a subicular cell shown in **Fig 6A** on the stem of the maze that were unidentifiable by the conventional rate–based method. One of the phase-based fields (red in **Fig 6A**) showed a larger amount of scene information than the rate-based field (0.32 > 0.06 for RDI_SCN–L_ and 0.43 > 0.25 for RDI_SCN–R_). The other phase-based field (green in **Fig 6A**) showed minimal information on the visual scene but carried more information on the choice response compared with the rate-based field (0.26 > 0.08 for RDI_CHC_). As illustrated by the neuronal examples in **Fig 6B**, some phase-based fields showed stronger rate remapping for scenes than for choices (cell 234–1–13–5 in CA1; cells 415–13–18–1, 415–13–10–1 in the subiculum), whereas other phase-based fields exhibited the opposite pattern (cell 561–1–14–6 in CA1; cells 232–6–19–1 and 415–10–19–2 in the subiculum). Furthermore, scene and choice information increased to a similar degree in some phase-based fields (**Fig 6B**, cell 234–3–19–6 in CA1; cells 415–11–19–1 and 232–4–18–3 in the subiculum).

We next investigated the extent to which task-related information carried by a single unit changed when the field detection protocol was changed from the rate-based to the phase-based method. For this purpose, if one cell showed multiple fields, the maximum RDI value was selected as the representative RDI of the cell (filled black dot in **Fig 6C**). Then, we calculated the “difference in RDI” (ΔRDI) by subtracting the representative RDI value of the rate-based protocol from the representative RDI value of the phase-based protocol for scene (ΔRDI_SCN_) and choice (ΔRDI_CHC_) information, respectively. Both RDI_SCN_ and RDI_CHC_ increased remarkably in the subiculum after theta phase–based field identification (T = 1582, *p* = 0.0002 for ΔRDI_SCN_; T = 2415, *p* < 0.0001 for ΔRDI_CHC_), but no significant increase was found in the CA1 (T = 365, *p* = 0.68 for ΔRDI_SCN_; T = 645, *p* = 0.47 for ΔRDI_CHC_; one-sample Wilcoxon signed rank test; **Fig 6D**). The RDI increases for subicular neurons were significantly higher than those for CA1 neurons for both visual scenes (Z = 3.26, *p* = 0.0011 for ΔRDI_SCN_) and choices (Z = 5.44, *p* < 0.0001 for ΔRDI_CHC_; Wilcoxon rank-sum test).

Since our phase-based field detection could separate the rate modulations that occurred separately in different fields (yet not identifiable based on the rate-based field detection; **Fig 6A**), we further examined if cells with multiple phase-based fields were associated with higher RDI values than cells with only single fields. For multi-field cells, RDI_SCN_ increased in both CA1 and subiculum (CA1, t_(45)_ = 3.40, *p* = 0.0014; subiculum, t_(165)_ = 4.10, *p* < 0.0001; **Fig 6E**), whereas RDI_CHC_ increased significantly only in the subiculum (CA1, t_(80)_ = 1.89, *p* = 0.065; subiculum, t_(50)_ = 6.63, *p* < 0.0001; **Fig 6F**). Cells with single fields did not show significant changes in their RDI values for both scene (CA1, t_(45)_ = 2.36, *p* = 0.020; subiculum, t_(165)_ = 0.52, *p* = 0.60) and choice (CA1, t_(80)_ = 2.37, *p* = 0.019; subiculum, t_(50)_ = 0.75, *p* = 0.46; Bonferroni-corrected one-sample *t* test; corrected α = 0.0125). A two-way ANOVA revealed that the magnitude of RDI changes was significantly different between multi-field and single-field cell groups (F_(3, 340)_ = 29.15, *p* < 0.0001 for RDI_SCN_; F_(3, 340)_ = 37.39, *p* < 0.0001 for RDI_CHC_). Specifically, ΔRDI was larger in multi-field cells than in single-field cells in both regions (CA1, t_(340)_ = 4.65, *p* = 0.0001; subiculum, t_(340)_ = 3.04, *p* = 0.0025 for RDI_SCN_, CA1, t_(340)_ = 2.90, *p* = 0.004; subiculum, t_(340)_ = 5.66, *p* < 0.0001 for RDI_CHC_). Note that there was a significant interaction between the effects of region and cell group for ΔRDI_CHC_ (F_(3, 340)_ = 4.69, *p* = 0.031) with a significant increase observed in subicular multi-field cell group only (t_(340)_ = 5.66, *p* = 0.0001; two-sample *t* test with Bonferroni-corrected α = 0.0125; **Fig 6F**).

For joint comparisons of changes in scene- and choice-based rate remapping, the differences in RDI_SCN_ and RDI_CHC_ of individual cells were marked as dots on a scatter plot (**Fig 6G**). As shown in the first and second quadrants of the scatter plot, RDI_SCN_ and RDI_CHC_ values increased jointly after applying the phase-based method in the subiculum compared to the CA1. Taken together, these results indicate that the theta phase–based field detection method is capable of identifying task-relevant information that would otherwise have been unidentifiable using the traditional rate-based field detection protocol. This trend was prevalent in the subicular multi-field cells and even in some CA1 multi-field cells.

### Subicular neurons represent scene and choice information more differentially through multiple phase-based fields compared to CA1 cells

**Fig 7:**
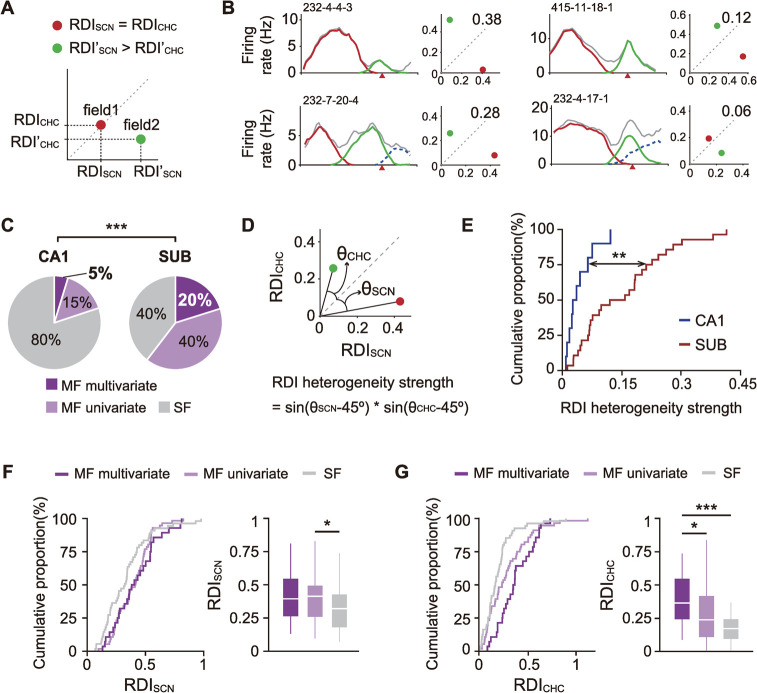
Scene and choice information are separately represented by multiple phase-based fields of subicular neurons. (**A**) Illustration showing the different relationships between RDI_SCN_ and RDI_CHC_ of example fields on the RDI scatter plot. Field 1 (red) near the diagonal shows the same amount of rate modulation for scene and choice information, whereas field 2 (green) located further away from the diagonal had much stronger rate remapping for scene than choice information. (**B**) Four examples of independent representations for scene and choice information for individual neurons. For each neuron, the left panel shows a linearized firing rate map (left) and the right panel shows an RDI scatter plot. Each phase-based field is color coded. Serial numbers above the rate map indicate cell IDs. Numbers on the scatter plots indicate the heterogeneity strength of RDI for different task variables. (**C**) Proportion of cells for which phase-based fields have independent representations for scene and choice information. ***p < 0.0001. (**D**) Illustration of how the strength of heterogeneous representations for scene and choice information is quantified. θ_SCN_ and θ_CHC_ indicate the angles between the diagonal and the vectors of the fields whose RDI_SCN_ or RDI_CHC_ is the maximum value. (**E**) Cumulative distribution of RDI heterogeneity strength for each region. **p < 0.01. (**F**–**G**) Cumulative proportion of subicular cells for RDI_SCN_ (F) and RDI_CHC_ (G). Bar graphs on the right side of each panel show RDI differences between subgroups within the subiculum. *p < 0.016, ***p < 0.0001. Data associated with this figure can be found in S1 Data file. MF, multi-field; RDI, rate difference index; SF, single-field; SUB, subiculum.

We further examined the functional significance of amplified task-related information (i.e., scene and choice) discovered by the phase-based method in subicular neurons compared with CA1 cells in our VSM task. If a place field showed the same amount of rate modulation for both scene and choice factors, the corresponding data point on the RDI scatter plot should be located on the diagonal (e.g., field 1 in **Fig 7A**). However, if the amount of rate remapping was influenced to a greater degree by one of the task-related factors, the data point should be located farther away from the diagonal (e.g., field 2 in **Fig 7A**, carrying more visual scene information than choice information). If a cell had multiple phase-based fields and each field represented either scene or choice information more strongly than the other, the cell was considered as coding *heterogeneous* task variables (i.e., scene and choice information) in the current study (**Fig 7B**).

A significantly larger portion of subicular neurons exhibited heterogeneous representations of task factors than CA1 neurons ($\chi_{\left(1\right)}^{2}$ = 18.65, *p* < 0.0001; chi-squared test; “MF multivariate” in **Fig 7C**). To test whether the heterogeneous representation of task factors occurred more strongly in subicular cells, we measured the angle between the diagonal and the vector of each phase-based field (θ_SCN_ and θ_CHC_; **Fig 7D**), and then calculated “heterogeneity strength” by multiplying the sine values of the angles. RDI heterogeneity was significantly stronger in the subiculum than in the CA1 (Z = 3.3, *p* = 0.001, Wilcoxon rank-sum test; **Fig 7E**).

Finally, we tested whether the amount of rate remapping differed between the following cell groups: multi-field cells with heterogeneous representations (MF multivariate), multi-field cells without such representations (MF univariate), and single-field cells (SF). Since the number of CA1 cells showing heterogenous representations for task variables was too small (*n =* 10) to obtain sufficient statistical power, tests were performed only within the subiculum. There were significant differences in both scene ($\chi_{\left(2\right)}^{2}$ = 7.79, *p* = 0.02, Kruskal–Wallis test; **Fig 7F**) and choice ($\chi_{\left(2\right)}^{2}$ = 19.44, *p* < 0.0001; **Fig 7G**) between the subgroups. Specifically, cells having multiple fields showed larger RDI_SCN_ values than those with single fields (MF multivariate versus SF: Z = 2.12, *p* = 0.034; MF univariate versus SF: Z = 2.5, *p* = 0.013; MF multivariate versus MF univariate: Z = 0.37, *p* = 0.7; Wilcoxon rank-sum test with Bonferroni correction; corrected *α* = 0.016). Moreover, subicular cells with heterogeneous representations exhibited significantly larger RDI_CHC_ values than other groups (MF multivariate versus SF: Z = 4.51, *p* < 0.0001; MF univariate versus SF: Z = 2.16, *p* = 0.031; MF multivariate versus MF univariate: Z = 2.4, *p* = 0.016). These findings indicate that subicular multiple fields identified based on the theta phase precessions of spikes did not represent task-relevant information uniformly. Instead, they carried heterogeneous task-related information in a more independent fashion compared with CA1 cells. Furthermore, such subicular cells represented task-related information more strongly in the VSM task compared to CA1 cells.

## Discussion

In the current study, we characterized the firing patterns of place cells in the CA1 and subiculum using both phase- and rate-based field detection methods. Our findings demonstrate that some of the broad place fields of subicular neurons can be parsed into multiple fields using the theta phase precession cycle. The newly discovered, phase-based place fields in the subiculum were more similar to those in CA1 in terms of spatial coding capacity and phase precession strength. However, unlike the case in the CA1, the neural representational strength of task-relevant information was significantly improved in the subiculum by the phase-based field detection method. Furthermore, our results suggest that firing for multiple fields by a single neuron may provide the subiculum with the unique function of representing different types of task-related information independently compared with the CA1.

### Underlying mechanisms of multiple cycles of theta phase precession and their associated place fields in the subiculum

One possible mechanism underlying the multiple cycles of theta phase precession and their associated place fields in the subiculum is convergent inputs from multiple place cells in the CA1 to a subicular cell. To our knowledge, whether a single subicular neuron is innervated by multiple CA1 place cells is still largely unknown. However, it has been reported that axon branches extending from a single CA1 pyramidal cell diverge to a very wide region within the subiculum, covering approximately 2 mm along the septotemporal axis [28] and one-third of the subiculum along the proximodistal axis [4] in rats. In addition, approximately 40% of CA1 pyramidal cells are known to send efferent projections to the subiculum [29]. Based on these anatomical characteristics, it is possible that a single subicular cell receives synaptic inputs from multiple CA1 pyramidal cells. If this is the case, a subicular place cell that receives inputs from multiple place cells in the CA1 whose firing peaks are located at distant locations may develop multiple place fields. Conversely, if multiple CA1 place cells sending projections to a single subicular place cell have overlapping place fields, then the subicular cell might exhibit a single broad firing field. Some prior studies may support these possibilities [30,31].

Another possibility is that the multiple fields of the subiculum might be based on inputs from cells in the medial entorhinal cortex, especially grid cells showing periodic firing fields and theta phase precessions. Some models have shown that theta phase precession in the CA1 could be derived from grid cells in the medial entorhinal cortex [32,33]. It has also been reported that temporal coding (including theta phase precession) in the CA1 is impaired by lesioning of the medial entorhinal cortex [33]. However, cells in layer 3 of the entorhinal cortex, which mainly project to the CA1 and subiculum, do not exhibit phase precession relative to theta rhythm [34]. Whether theta phase precession in the subiculum is inherited from grid cells in the medial entorhinal cortex remains to be investigated.

Lastly, there is the possibility that cells in the subiculum might be influenced by multiple sources of theta rhythm—one from an extracellular source and another generated intrinsically. Specifically, some previous studies have proposed an interference model as the mechanism for theta phase precession in the CA1 [25,35,36]. According to this model, there is an intrinsic theta oscillator within pyramidal cells that causes theta phase precession while maintaining a frequency that can be different from that of the extracellular theta rhythm. This model is supported by experimental evidence showing that pyramidal cells in the dorsal hippocampus show higher intrinsic oscillation frequencies than those in the ventral hippocampus, resulting in smaller place fields in the dorsal hippocampus [37]. Experimental evidence for the presence of an intrinsic theta oscillator in the subiculum has not been reported. However, because principal cells in the subiculum have denser recurrent connectivity than those in the CA1 [38–40], it is possible that cells in the subiculum can generate local rhythms intrinsically. Notably, a recent study reported that an atypical type of sharp–wave ripple occurs in the subiculum, independent of traditionally known CA3-originating sharp–wave ripples [41], an observation that may support the possibility that subicular neurons intrinsically generate their own local oscillations.

### Clustering algorithm for identifying multiple cycles of theta phase precession and their associated place fields

A previous study by Maurer and colleagues [24] demonstrated that the partially overlapping place fields of a single cell in the CA1 could be segmented by manually drawing boundaries around the spikes belonging to individual cycles of theta phase precession on the position phase scatter plot. Further improving this strategy, Kim and colleagues [10] developed an automated algorithm that constructed a phase position firing rate map from normalized phase position plots of rat occupancy and then defined place fields based on detection of local maxima. However, there were challenges to adopting this previous protocol in the current study. First, these authors used a behavioral paradigm in which rats ran along a track in the absence of environmental change or mnemonic task demand, whereas in the current study, rats performed a mnemonic task in which they were required to associate different scenes with discrete behavioral choices. Our previous study showed that firing rates of subicular cells are modulated in relation to task-related information (i.e., scene and choice) [11]. In that case, even if a field had a high firing rate in one condition, it might show a low firing rate in the other condition. Accordingly, in the overall firing rate map obtained by averaging the firing rates for all conditions, the high firing rate under one specific condition is likely to be canceled out by that under the other condition, making it difficult to definitively establish peak firing and field boundaries when defining a place field. Second, the length of the linear track used in the current study was relatively short, possibly resulting in more overlap between place fields and thus creating difficulties in setting an appropriate threshold for segmenting individual fields on the phase position firing rate map.

Therefore, to eliminate the risk of not being able to find potential task-related firing fields, we adopted the clustering algorithm DBSCAN, which can be applied to the raw phase position spiking plot without normalizing for occupancy. We chose this algorithm for several reasons. First, when sampled sufficiently, spikes tend to occur at the most preferred location within a place field with highest probability and then gradually diminish as the distance from the field center increases (i.e., Gaussian-like distribution). Because of its density-based algorithmic nature, the DBSCAN algorithm is suitable for finding clusters when data points exhibit such distributions. Second, DBSCAN has the advantage of robustly detecting outliers, which enabled us to process continuous and spontaneous firing activities of subicular fields. Furthermore, DBSCAN does not require an experimenter to predetermine the number of clusters. Finally, the DBSCAN algorithm does not limit cluster shape, so it can flexibly find clusters in a complex data set.

### Functional significance of the more independent representation of scene and choice information in the subiculum than the CA1

The spatial firing properties of subicular neurons are different from those of CA1 cells. This unique nature of the subiculum has been attributed to signals from outside the hippocampal formation, including those related to movement and head direction of the thalamic region [42]. However, task-related information such as scene and choice information in the subiculum should be influenced by inputs from the dorsal CA1, as demonstrated in our previous studies [22,43]. This possibility is supported by findings of the current study showing significantly enhanced rate modulation for such task-relevant information, as only the group of spikes constituting a single cycle of theta phase precession was extracted for measuring the representational strength of task-related information.

The enhancement of task-related signals in the subiculum allowed us to investigate the functional roles of subicular cells in the VSM task compared with our previous study in which we relied on the traditional rate-based field detection algorithm [11]. Remarkably, some subicular neurons carried scene and choice information separately in their subfields. This phenomenon could arise if different CA1 cells, each carrying one type of task-related information more strongly than the other, send their outputs to a single subicular neuron. Subicular neuron could then facilitate associative learning by representing different types of information concurrently so that downstream structures (e.g., prefrontal cortex) receive more associative information between the critical task variables. Note that a subicular cell tends to represent different task-related information in separated fields associated with distant locations, but not conjunctively in one field. The different types of information represented by the separate fields of a subicular cell might then be transmitted into identical target regions nearly simultaneously with a certain phase relationship, potentially contributing to organized actions based on hippocampal memory representations.

### Functional subclasses of neurons in the subiculum may play key roles in hippocampal-dependent action in a visual contextual memory task

Numerous studies have suggested the presence of anatomical and physiological subpopulations in the subiculum. Specifically, it has been reported that afferent and efferent projections of the subiculum are organized topographically along the proximodistal axis [4–6,8,13,28,44–46], as the proximal and distal parts of the subiculum are clearly divided according to gene expression in principal cells [6,47]. In addition to these anatomical subdivisions, in vitro physiological studies have reported 2 types of intrinsic firing for subicular principal cells—bursting and regular spiking [48–50]—and have shown that these cells exhibit a unique distribution gradient along the proximodistal axis [40,51,52]. These 2 classes of cells are modulated differently by sharp–wave ripples of the CA1 and have different intrinsic connectivity [38]. Furthermore, a series of recent in vivo studies identified subpopulations in the subiculum with different spatial firing characteristics [29,53–55].

Our physiological results also suggest that there are functionally different subclasses of neurons within the subiculum. In our previous paper, subicular cells with a single broad field showed a “schematic” firing pattern that depended on the cognitive structure of the task; we speculated that this firing pattern serves to mediate contextual behavior by representing the discrete region associated with critical epochs of the task [11]. That is, cells in the CA1 and subiculum represent specific location information and an epoch-based region, respectively. On the other hand, subicular cells with multiple focal fields are thought to contribute to associative memory by subsequently—but almost concurrently—transmitting different types of task-relevant information to downstream structures where choice-related actions and decisions occur.

Taken together, our findings indicate that the subiculum may support visual contextual behavior in space through 2 processes, each driven by a distinctive neuronal class. Information regarding context and future path leading to the goal location, separately recognized at local and distant place fields of the CA1, could be integrated by subicular cells by multiple phase-based fields and transmitted to downstream areas. In particular, such information processing in the subiculum could be critical for converting contextual information into a goal-directed action signal. At the same time, subicular cells with a broad single field may represent the area in which all locations are associated with a common task-related variable, such as a specific visual scene or choice response. A recent study showing that CA1-projecting subicular cells receive direct inputs from the visual cortex and send their projections to some critical regions (e.g., perirhinal cortex, CA1) may also support the functional significance of the subiculum in the visual contextual behavioral task [56].

## Materials and methods

### Subjects

Male Long–Evans rats (*n =* 5) were used in the current study. Food was restricted to maintain rats’ body weights at 350 to 400 g (85% of free-feeding weight), and water was made available ad libitum. Rats were individually housed under a 12-h light/dark cycle. All experimental protocols followed the Laboratory Animal Act of Korea and were approved by the Institutional Animal Care and Use Committee of the Seoul National University (SNU–200504–3–1).

### Behavioral task

Detailed descriptions of our experimental procedures, including the VSM task, the apparatus, and surgery (**Fig 1A**), are available in our previous study [11]. Briefly, the rat was located in a start box before a trial began. The experimenter started the trial by opening the door of the start box, which also triggered presentation of a patterned visual stimulus (i.e., visual scene) in an array of 3 adjacent LCD monitors surrounding the choice–arm region of the T-maze. The rat then entered and ran along the stem of the T-maze (stem, 73 × 8 cm; arms, 38 × 8 cm) and was required to turn left or right at the end of the stem (“choice point”) in association with the visual scene. The rat obtained a quarter piece of cereal reward (Froot Loops, Kellogg’s) from the food well at the end of the correct arm, but no reward was given if it entered the wrong arm. Four visual scenes (zebra, bamboo, pebbles, mountains) were used. In all sessions, zebra stripes and bamboo patterns were associated with the left arm, and pebbles and mountain patterns were associated with the right arm; within a session, the 4 visual scenes were presented in a pseudorandom sequence.

During the presurgical training period, the rat was initially trained with a pair of scene stimuli (zebra versus pebbles or bamboo versus mountain, counterbalanced for rats) until it reached the performance criterion for each pair (≥75% correct for each scene for 2 consecutive days; 40 trials/session). It took approximately 2 weeks (13.4 ± 0.9 sessions, mean ± SEM) for rats (*n =* 5) to reach the performance criterion for both scene pairs. Afterwards, a hyperdrive carrying 24 tetrodes (+3 reference electrodes) was surgically implanted in the right hemisphere to cover 3.2 to 6.6 mm posterior to bregma and 1 to 4 mm lateral to the midline. After 1 week of recovery, the rat was retrained until it reached presurgical performance levels, during which time the tetrodes were lowered into the subiculum and CA1 by 40 to 160 μm daily. Thereafter, the main recording sessions (123 ± 6 trials/session, mean ± SEM) began, and the 4 scene stimuli were presented in an intermixed fashion during sessions.

### Electrophysiological recording and histological procedures

Single unit spiking activity and local field potentials (LFPs) were recorded from the dorsal CA1 and subiculum. Neural signals were transmitted to the data acquisition system (Digital Lynx SX; Neuralynx) through a headstage connected to the EIB board and tethered via a slip–ring commutator on the ceiling. Neural signals from tetrodes were amplified 1,000 to 10,000 times and sampled at 32 kHz. Spiking data were acquired by filtering at 600 to 6,000 Hz. LFPs were obtained by filtering the same signals at 0.1 to 1,000 Hz. After completion of all recording sessions, electrolytic lesions (10 μA current for 10 s) were made to mark the tip positions of the tetrodes. Twenty-four hours after electrolytic lesioning, the rat was killed by inhalation of an overdose of CO_2_ and perfused transcardially. Brain tissue was stained using thionin or Timm’s method for Nissl substances (see details in our previous paper [11]; **Fig 1C**).

The anatomical boundaries of the CA1 and subiculum were determined based on the rat brain atlas [57]. Tetrodes located in the transition area between the CA1 and subiculum were excluded. To quantitatively describe the proximodistal positions of the recording tetrodes, we measured the linearized length of the cell layer in the CA1 and subiculum—specifically, the distance between the most distal to the proximal end along the curved pyramidal cell layer in a given section—using image processing software (ImageJ; NIH). Recording positions across rats were normalized by selecting a median value among the linearized lengths of the pyramidal cell layers of the CA1 and subiculum in all rats, and the ratio between the CA1 and subiculum was obtained (subiculum:CA1 = 0.36:0.64). The relative positions of tetrode tips within each region were then calculated (**Fig 1D**).

### Extraction of outbound running epochs

Before proceeding with a set of analyses based on spiking data in relation to their theta phases, we extracted only those epochs associated with outbound journeys (from the start box to either left or right food well). To facilitate theta rhythm–related analyses, we calculated the instantaneous running speed so as to include epochs in which rats ran at a reasonable speed. To this end, we interpolated linearized position data to compensate for vacancies caused by rat head movements and/or tether interference. Next, outlier data points were suppressed using a locally weighted robust regression. Then, the instantaneous running speed, calculated by dividing the length of 3 consecutive data points by the duration of time, was assigned to the middle point of the three. The average running speed was 35.3 cm/s in all sessions for all rats. Spikes that occurred when running speed was greater than 20 cm/s were used in this study. If the latency from the start box to the food well was longer than 6 s, that trial was discarded.

### Spiking data analysis

#### Unit isolation

Single units were isolated manually using both commercial software (SpikeSort3D; Neuralynx) and a custom-written program (WinClust) based on the waveform parameters, peak amplitude, energy, and peak-to-valley latencies. Fast spiking neurons (mean firing rate ≥ 10 Hz; width of the average waveform < 325 μs) were excluded from analysis. The same criteria from our previous study (Lee and colleagues, 2018) were used to evaluate unit isolation quality, with the additional criterion that the number of spikes during running epochs of outbound journeys on the track should be greater than 50 (CA1, *n =* 270 units; subiculum, *n* = 151 units).

#### Detection of firing rate–based place fields

Position data acquired during outbound running epochs were first linearized by scaling down using 2-cm spatial bins. The choice point—that is, the point where the rat’s position data diverged between left and right choice trials—was determined by detecting the spatial bin with a statistical difference between left and right position traces (two-sample *t* test). Then, a linearized firing rate map was constructed by dividing the number of spikes by the number of position data points in individual spatial bins. Boundaries of a firing field were defined as the first spatial bin at which the firing rate dropped below 33% of the peak firing rate for 2 consecutive bins. If a local peak exceeding 50% of the maximum peak firing rate was found outside the predetermined firing field, it was considered as the peak of a possible subfield, and the boundaries of the subfield were found using the same algorithm. After defining the field boundaries, a firing field was identified as a place field when the peak firing rate within the field exceed 1 Hz and the spatial information score of the field was greater than 0.5. The spatial information score was computed as follows [58]:

$$\mathrm{Spatial}\;\mathrm{information}\;\mathrm{score}\;(\mathrm{Rate}\;\mathrm{information})=\sum_{i}p_{i}\frac{\lambda_{i}}{\lambda }\log_{2}\frac{\lambda_{i}}{\lambda }(\mathrm{bits}/\mathrm{spike}),$$

where *i* denotes the spatial bin, *p_i_* is the occupancy rate in the *i*^th^ bin, *λ_i_* is the mean firing rate in the *i*^th^ bin, and *λ* is the overall mean firing rate. The mean firing rate were obtained from the raw rate maps. For display, the rate maps were smoothed using the adaptive binning method. Another spatial information score based on spiking theta phases was obtained using the following formula [59,60]:

$$\mathrm{Spatial}\;\mathrm{information}\;\mathrm{score}\;(\mathrm{Phase}\;\mathrm{information})=\sum_{K\geq 0}P_{K|x_{i}}\log_{2}\frac{P_{K|x_{i}}}{P_{k}}\;(\mathrm{bits}/\mathrm{cm}),$$

where $P_{K|x_{i}}$ is the conditional probability of observing rate K in position x_i_, and P_k_ is the probability of observing a rate k in all position bins. Additional spatial coding metrics such as spatial sparsity and selectivity per cell were calculated as follows [26]:

$$Sparsity=\frac{{\left(\sum p_{i}\lambda_{i}\right)}^{2}}{\left(\sum p_{i}\lambda_{i}^{2}\right)},\;Selectivity=\frac{max(\lambda_{i})}{\lambda },$$

where the same symbols were used as in the formula for calculating spatial information above.

### LFP analysis

#### Tetrode selection

To align baseline offsets, we down-sampled LFPs from 32 kHz to 2 kHz and filtered them at 3 to 300 Hz using a zero-phase bandpass filter (third-order Butterworth filter with the *filtfilt* function in MATLAB). LFP traces from running epochs were then visually inspected to exclude tetrodes whose voltage traces exceeded the maximum value (3,000 μV) of the analog–digital converter or artifacts such as bumping noises. Spiking phases in relation to theta rhythm were analyzed by obtaining a power spectral density (PSD) function using a multi–taper method (Chronux ToolBox; MATLAB) and then selecting reference tetrodes with the strongest power in the high theta band (7 to 12 Hz) for individual sessions and regions. The frequency range of the theta band was set so as to include the most prominent peak at 8 Hz in the mean PSD function during the outbound journey and to minimize bumping noises that usually occurred at less than 7 Hz. LFPs recorded from the CA1 and subiculum were used for spiking phase analyses of single units in the corresponding regions.

#### Spiking theta phases

LFPs from reference tetrodes were filtered in the theta range (7 to 12 Hz) using a zero-phase bandpass filter, followed by application of a Hilbert transform to decompose filtered LFPs into amplitude and phase information. Spiking-phase relationships were examined by plotting instantaneous theta phases and rat’s linearized positions at time points when spikes occurred in a 2D space (phase position plot).

#### Identification of theta phase–based place fields using DBSCAN

To define a cluster of spikes that shared the same spiking-phase relationships, we adopted a well-known clustering algorithm called the DBSCAN suggested by Ester and colleagues [61]. DBSCAN is a density-based, nonparametric algorithm that gathers data points in close proximity while excluding distant or sparsely located points as noise. In DBSCAN, it is not necessary to specify the number of clusters in advance. Still, some parameters must be predetermined to run the algorithm, such as the distance (ε) and the minimum number of points within a distance (N_min_). Specifically, if the number of data points at a distance ε from a point is greater than N_min,_ including itself, the point is defined as a core point of a cluster. If another point contains the core point within distance ε but does not satisfy N_min_, it is defined as a border point. If there is no core point and N_min_ is not satisfied, the point is defined as a noise point. In our study, clusters on the position phase plot were captured by the DBSCAN algorithm to find theta phase–based place fields (“TP-based place fields”). To avoid detecting spurious sparse clusters, we restricted DBSCAN parameters to the following ranges: distance (ε) < 8 cm; N_min_ ≥ 10; and total number of spikes in a cluster ≥ 30. The parameters were determined manually in those ranges so that the number of clusters in a cell was greater than the number of local maxima in linearized firing rate maps. Biased clustering caused by experimenter subjectivity was prevented by performing cross-validation with 3 additional experimenters who did not participate in analyses of the current data sets. After cross-validation, cells with invalid clustering were excluded from the analysis based on the following: (1) DBSCAN parameters not satisfied (subiculum, *n =* 21; CA1, *n* = 24); (2) insufficient spikes (subiculum, *n* = 17; CA1, *n* = 30); or (3) irregular cluster shape (subiculum, *n* = 16; CA1, *n* = 6).

#### Quantification of theta phase precession

After identification of individual place fields using theta phases, the slopes of theta precession were measured by fitting individual spike clusters to circular–linear regression lines [27]. A circular–linear correlation was also applied to determine if the phase shift was significant (Toolbox for circular statistics with MATLAB) [62]. Theta phase precession of a place field was considered significant if the following criteria were met: (1) range of phase shift ≥90°; (2) slope of regression line <0; and (3) *p*-value of circular–linear correlation ≤0.05. The onset phase and the range of theta phase precession were only obtained from the phase-based fields with significant phase precession. The onset phase was defined as the starting phase of the circular–linear regression line at the beginning of the field. The range of phase shift was set as the phase difference between the starting phase and ending phase of the regression line.

### Analysis of rate remapping

To measure the amount of rate modulation between firing rate maps associated with different trial conditions (i.e., scene stimulus or choice response), we obtained an RDI by calculating an absolute value of *Cohen’s d*:

$$Rate\;difference\;index=|\frac{mean({FR}_{1})-mean({FR}_{2})}{std({FR}_{1},{FR}_{2})}|,$$

where *FR*_1_ and *FR*_2_ denote the in-field firing rates of individual trials associated with different conditions. Cohen’s d measure was used in the current study because it includes a term for standard deviation in its denominator, which controlled the confounding effect that might be induced by the differential variability of in intrinsic neuronal firing between the CA1 and subiculum (**S3 Fig**). With respect to RDI for scene stimuli, 2 RDI values were obtained from 2 pairs of scenes associated with the left or right choice arm (RDI_SCN–L_ and RDI_SCN–R_, respectively), then a maximum value was chosen as a representative scene-based RDI of a cell (RDI_SCN_). RDI for choice response (RDI_CHC_) was measured by calculating the difference in firing rates between left and right choice trials. For calculation of RDI_CHC_, only the firing rate maps associated with the area between the start point (i.e., start box door) and the choice point of the stem of the T-maze were used because the rat’s position traces after the choice point diverged between the left-choice and right-choice trials. Since RDI_SCN_ was originally calculated from firing rate maps associated with the same choice arm, all spiking activities on the maze including those that occurred after the choice point were used. However, if a spatial bin with a firing rate less than 75% of the peak firing rate was located in one arm of the maze, the field was considered as an arm field and was excluded from the RDI analysis. Accordingly, cells with only single arm fields were excluded as well.

To quantify the change in RDI (ΔRDI) within a cell after detecting its field based on theta phase–based method, the largest RDI value for RDI_SCN_ and RDI_CHC_ was chosen as the cell’s representative RDI value. The representative RDI_SCN_ and RDI_CHC_ could be selected from the cell’s different fields except arm fields. If a cell had only a single field, then RDI of that field was equated with its representative RDI. Then, ΔRDI was calculated by subtracting the representative RDI value of the rate-based protocol from the representative RDI value of the phase-based protocol. The same protocol was used in both rate- and phase-based methods. Cells having place fields by both rate- and phase-based methods were used (CA1, *n =* 212; subiculum, *n* = 132).

For analysis of the heterogeneity of RDI values, the angles between the diagonal line and the vectors of fields with maximum RDI_SCN_ or RDI_CHC_ were obtained. Then, the product of their sine values, defined as the strength of RDI heterogeneity, was obtained. This measurement was adopted because it had the characteristic that its value approached zero as any one of the fields came close to the diagonal. Task heterogeneity could not be measured for MF univariate cells in our study because the 2 fields were located on the same side of the diagonal for those cells. In this analysis, cells having place fields by phase-based methods were all used (CA1, *n* = 212; subiculum, *n* = 139).

To confirm the ability of RDI to control the variability in intrinsic firing, rate modulation index (RMI) was also obtained for each cell (S3 Fig). In the same way as the RDI calculation method above, RMI was calculated by obtaining the mean firing rate associated with each scene or choice condition within individual phase-based fields. Because the denominator of the formula does not include a pooled standard deviation, RMI is regarded as an uncontrollable measurement of firing variability within a cell.


$$Rate\;modulation\;index=|\frac{mean({FR}_{1})-mean({FR}_{2})}{mean({FR}_{1})+mean({FR}_{2})}|.$$

### Statistical analysis

Both the behavioral and neural data were analyzed using nonparametric statistical tests with the level of statistical significance set at α = 0.05 unless noted otherwise. Testing for statistical significance was two-sided, except when testing significance against a specific known value. For example, a one-sample Wilcoxon signed rank test was used to compare the behavioral performance for different scene stimuli against our performance criterion of 75% and to test whether the differences in RDI_SCN_ or RDI_CHC_ between field identification methods were significantly above zero. The proportional differences in cell types between 2 regions or 2 methods were tested using a chi-squared test. The differences in slope and strength of theta phase precession were examined by two-way mixed ANOVA with region as a between-subject and method as a within-subject factor, but an unpaired two-sample *t* test with Bonferroni correction was used for post hoc test because the number of observations for the within-subject factor was different across cells. A two-way ANOVA was conducted to compare the regions and cell groups (MF cells and SF cells) with respect to ΔRDI_SCN_ and ΔRDI_CHC_, and an unpaired two-sample *t* test with a Bonferroni correction was used for post hoc tests. Differences in RDI_SCN_ and RDI_CHC_ among cell types (i.e., MF multivariate, MF univariate and SF) were assessed using a Kruskal–Wallis test, with the application of the Bonferroni-corrected Wilcoxon rank-sum test for post hoc comparisons.

## Supporting information

S1 Fig Categorization of cells after theta phase–based field detection. Population rate maps of CA1 and subicular cells that are grouped into SF cells or MF cells after the application of the theta phase–based field detection method. MF, multi-field; SF, single-field.
(TIF)

S2 Fig Basic firing properties of CA1 and subicular phase-based fields are comparable. (**A**) A 3D scatter plot consisting of phase information, rate information, and mean firing rate calculated by using the entire spiking activities associated with outbound journey in a given cell. Dots indicate cells in the CA1 (blue) and subiculum (red). (**B**) Same as in (A), but the values are obtained from in-field firing activities of phase-based subfields. Dots indicate individual phase-based fields. Data associated with this figure can be found in S1 Data file.
(TIF)

S3 Fig RDI was used to control within-cell variability of intrinsic firing. (**A**) Illustration of the RDI (or Cohen’s d) reflecting within-cell variability. The distributions of firing rates associated with either left trials (gray) or right trials (green) are drawn as histograms. Two example neurons show similar amounts of difference in their mean firing rates between the trials associated with the left and right choices. However, RDI values were different due to the difference in pooled standard deviations between the 2 cells. (**B**) Comparison of RMI with RDI with respect to correlation with firing rates. Dots indicate individual cells of the CA1 and subiculum, and red lines are linearly fitted lines. Correlation coefficient is indicated on each plot. Note that RMIs show stronger correlations with mean firing rates than RDI values. Data associated with this figure can be found in S1 Data file. RDI, rate difference index; RMI, rate modulation index.
(TIF)

S1 Data Data set that underlies the results and figures of the paper. (1B) Behavioral performance data used in Fig 1B. (1D) Normalized anatomical positions of CA1 and subicular neurons along the proximo-distal axis that used in Fig 1D. (2D–E, S2A) Basic firing properties obtained from individual cells using the entire outbound spiking activities. (2C, 2F, 5A–H, S2B) Basic firing properties and theta phase precession properties obtained from individual rate- and phase-based fields. (6D–G) RDI changes per cell between the rate- and phase-based methods. (7C–G) RDI heterogeneity strength of MF multivariate cells and RDI values of subicular neurons. (S3A) Mean firing rates of individual trials of 2 example CA1 neurons sorted by choice condition. (S3B) RDI and RMI values obtained from individual phase-based fields. MF, multi-field; RDI, rate difference index; RMI, rate modulation index.
(XLSX)

## Abbreviations

DBSCAN: Density-Based Spatial Clustering with Applications of Noise
LFP: local field potential
MF: multiple fields
NF: no field
PSD: power spectral density
RDI: rate difference index
RMI: rate modulation index
SF: single field
SUB: subiculum
TPP: Theta phase precession
VSM: visual scene memory
