# Supplementary figures and images for: Subicular neurons represent multiple variables of a hippocampal-dependent task by using theta rhythm
Source: PLoS Biol. 2022 Jan 31;20(1):e3001546. doi: 10.1371/journal.pbio.3001546 (PMC8830791; doi:10.1371/journal.pbio.3001546)

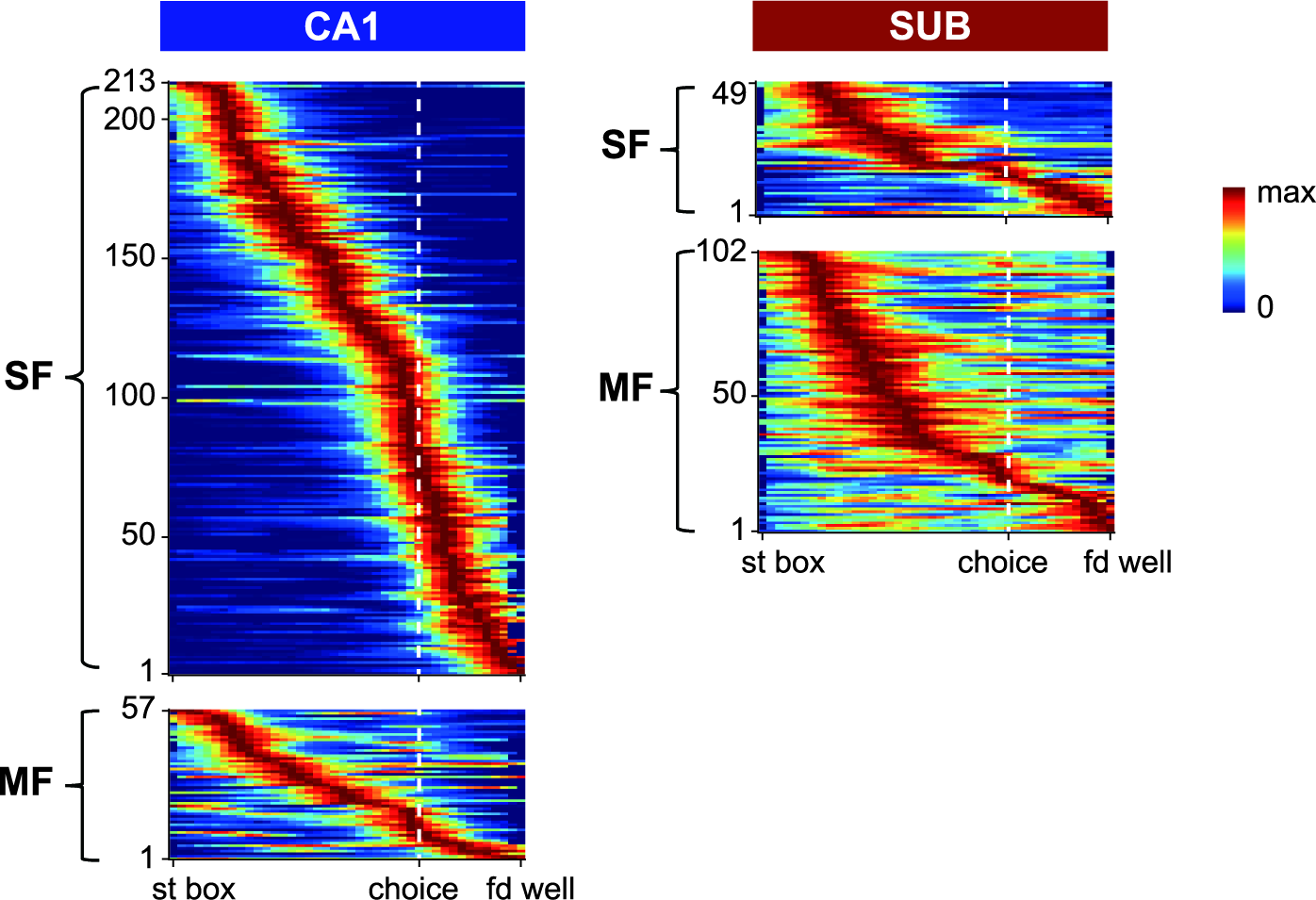

Supplement: S1 Fig — Population rate maps of CA1 and subicular cells that are grouped into SF cells or MF cells after the application of the theta phase–based field detection method. MF, multi-field; SF, single-field. (TIF) [file pbio.3001546.s001.tif]

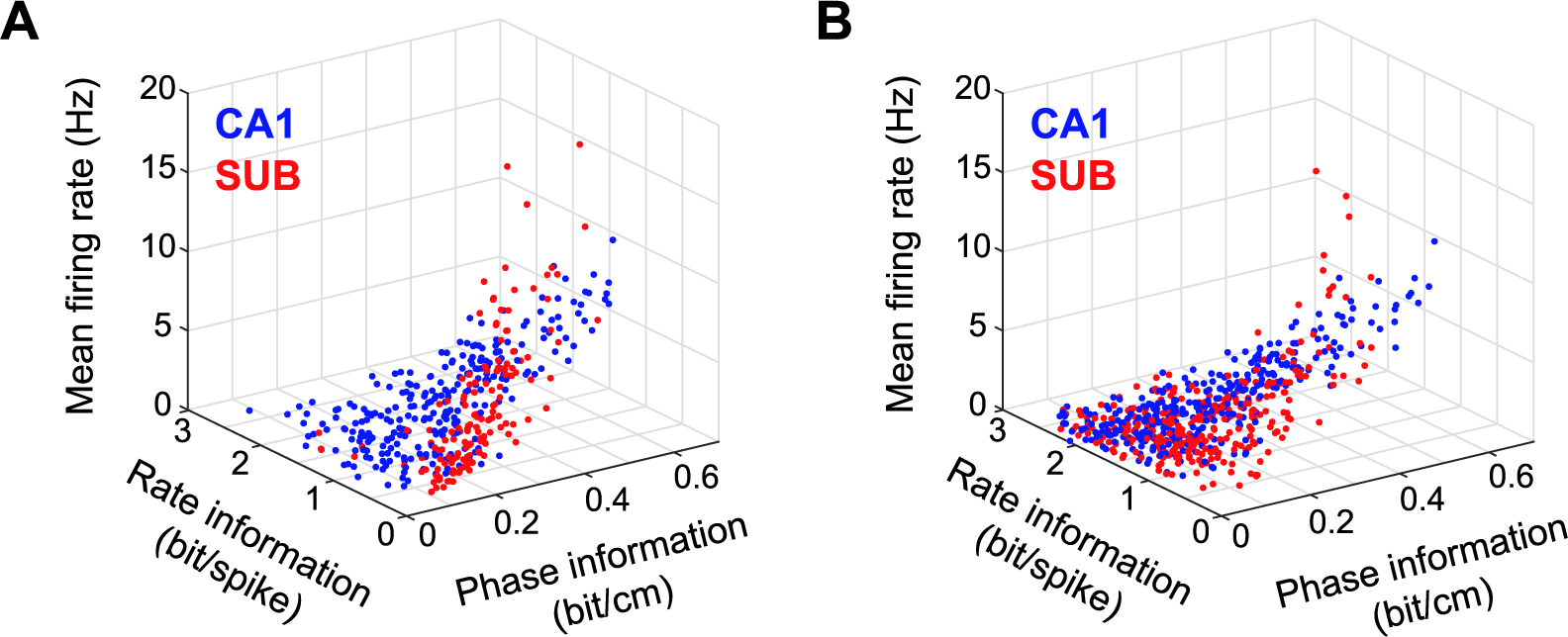

Supplement: S2 Fig — (A) A 3D scatter plot consisting of phase information, rate information, and mean firing rate calculated by using the entire spiking activities associated with outbound journey in a given cell. Dots indicate cells in the CA1 (blue) and subiculum (red). (B) Same as in (A), but the values are obtained from in-field firing activities of phase-based subfields. Dots indicate individual phase-based fields. Data associated with this figure can be found in S1 Data file. (TIF) [file pbio.3001546.s002.tif]

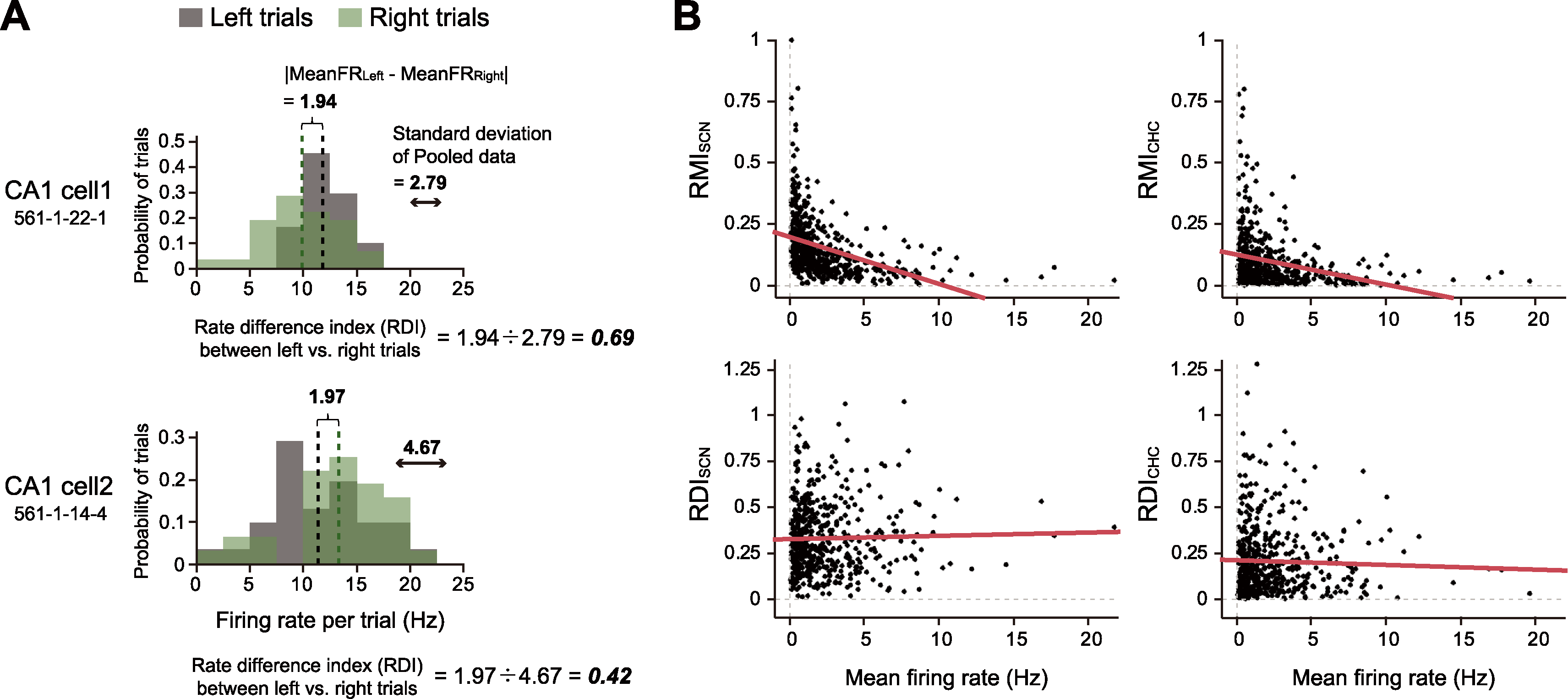

Supplement: S3 Fig — (A) Illustration of the RDI (or Cohen’s d) reflecting within-cell variability. The distributions of firing rates associated with either left trials (gray) or right trials (green) are drawn as histograms. Two example neurons show similar amounts of difference in their mean firing rates between the trials associated with the left and right choices. However, RDI values were different due to the difference in pooled standard deviations between the 2 cells. (B) Comparison of RMI with RDI with respect to correlation with firing rates. Dots indicate individual cells of the CA1 and subiculum, and red lines are linearly fitted lines. Correlation coefficient is indicated on each plot. Note that RMIs show stronger correlations with mean firing rates than RDI values. Data associated with this figure can be found in S1 Data file. RDI, rate difference index; RMI, rate modulation index. (TIF) [file pbio.3001546.s003.tif]
